# Expression and secretion of *Aspergillus fumigatus* proteases are regulated in response to different protein substrates

**DOI:** 10.1016/j.funbio.2012.07.004

**Published:** 2012-09

**Authors:** Edward Farnell, Karine Rousseau, David J. Thornton, Paul Bowyer, Sarah E. Herrick

**Affiliations:** a Respiratory Research Group, School of Translational Medicine, Faculty of Medical and Human Sciences, University of Manchester, Stopford Building, Manchester M13 9PT, UK; b Wellcome Trust Centre for Cell-Matrix Research, Faculty of Life Sciences, University of Manchester, Michael Smith Building, Manchester M13 9PT, UK; c Respiratory Research Group, School of Translational Medicine, Faculty of Medical and Human Sciences, University of Manchester, Education and Research Centre, Wythenshawe Hospital, Manchester M23 9LT, UK

**Keywords:** Allergen, Culture, Fungal, Growth, Proteases, Secretion

## Abstract

The ubiquitous filamentous fungus *Aspergillus fumigatus* secretes a number of allergens with protease activity and has been linked to a variety of allergic conditions such as Severe Asthma with Fungal Sensitization (SAFS) and Allergic Bronchopulmonary Aspergillosis (ABPA). However, it is unclear which allergen proteases are being secreted during fungal invasion and whether the local biological environment regulates their expression. Understanding the dynamic expression of allergen proteases during growth of *A. fumigatus* may lead to further characterisation of the pathogenesis of these disorders as well as improved standardisation in the commercial production of these allergens. Secretion of proteases during germination and early growth of *A. fumigatus* was investigated in response to various complex protein sources (pig lung homogenate, mucin or casein). Protease inhibitor studies demonstrated that *A. fumigatus* (AF293 strain) secretes predominately serine proteases during growth in pig lung based medium and mainly metalloproteases during growth in casein based medium but suppressed protease secretion in unmodified Vogel's minimal medium and secreted both types in mucin based medium. Analysis of gene transcription and protein identification by mass spectrometry showed that the matrix metalloprotease, Mep/Asp f 5 and the serine protease, Alp1/Asp f 13, were upregulated and secreted during growth in pig lung medium, whereas Alp1 was predominately expressed and secreted in mucin based medium. In casein medium, the matrix metalloprotease, Lap1, was also upregulated and secreted in addition to Mep and Alp1. These findings suggest that *A. fumigatus* is able to detect different complex proteins available as substrates in its environment and regulate protease secretion accordingly. There is a requirement for the standardisation of *A. fumigatus* allergen extracts used both in clinical diagnosis of *A. fumigatus* allergy and in research studies.

## Highlights

► *Aspergillus fumigatus* produces allergens with protease activity. ► Growth on protein based substrates induces the secretion of proteases. ► Mainly serine proteases secreted in response pig lung homogenate or mucin media. ► Predominately MMP secreted in response to casein media. ► *Aspergillus fumigatus* can sense and respond to changes in growth environment.

## Introduction

*Aspergillus fumigatus* (*Af*) is a filamentous fungus that is ubiquitous within the environment. In compost, it plays an important role in the breakdown of organic material and the recycling of carbon and nitrogen whereas in the human host, it is involved in a number of diseases associated with immune perturbations. For instance, in neutropenic individuals, *Af* is a frequent opportunistic pathogen and causative agent in invasive aspergillosis (Bodey & Vartivarian 1989). In atopic individuals, *Af* is involved in allergic lung diseases such as Severe Asthma with Fungal Sensitization (SAFS) and Allergic Bronchopulmonary Aspergillosis (ABPA) (Denning *et al.* 2006).

Exposure to fungi is ubiquitous although the precise allergens that individuals are exposed to may be different depending on the source of the allergic material. It has been suggested that, unlike exposure to allergens from other sources, such as house dust mite, chronic exposure to fungal allergens may also occur during colonisation of the host lung by fungi and that the products of germinating conidia and hyphae may be responsible for allergy and the development of asthma (Denning *et al.* 2006). Such theories have been supported by a number of studies where colonisation of the lung by conidia of *A*. *niger* in a murine model of airway allergy results in the development of atopy and allergy (Porter *et al.* 2009). Of note, the majority of allergens from nonfungal sources that have been linked to asthma also show protease activity (Reed 2007). To date, over 25 allergens have been identified from *Af*, a number of which have protease activity (Bowyer & Denning 2007) and demonstrate biological function. Furthermore, exposure of lung alveolar and bronchial epithelial cells to germinating *Af* conidia or crude *Af* culture extract containing the metalloprotease allergen, Mep (Asp f 5) and serine protease allergen, Alp 1 (Asp f 13), has been shown to cause protease-dependent release of proinflammatory cytokines, IL-6, and IL-8 (Tomee *et al.* 1997; Kauffman *et al.* 2000; Bellanger *et al.* 2009). Additionally, Alp1 has been shown to act in a protease-dependent manner, as an adjuvant during sensitisation to ovalbumin in a murine model of allergy (Kheradmand *et al.* 2002). These studies would suggest the involvement of *Af* allergen proteases in allergic airway disease, however the conditions regulating allergen protease secretion are not clear.

Previous work has shown that *Af* regulates the expression of allergen proteases during growth under different culture conditions including oxidative stress and anoxia (Fraczek *et al.* 2010). Others have shown that *Af* secretes different sets of proteases depending on the pH of the growth medium (Sriranganadane *et al.* 2010). *Af* has also been shown to secrete allergen proteases when grown in liquid culture with complex proteins such as collagen and elastin as the sole carbon and nitrogen source (Monod *et al.* 1991; Moutaouakil *et al.* 1993; Gifford *et al.* 2002; Bergmann *et al.* 2009) and *in vivo* during colonisation of mouse lung (Monod *et al.* 1991; Moutaouakil *et al.* 1993). *Af* is only able to assimilate amino acids and short peptides *via* membrane transporters, therefore the secretion of proteases to breakdown large peptide sequences is likely to be critical to allow growth in environments dominated by protein as the sole carbon and nitrogen source such as the lung.

Although proteases have been shown to be produced by *Af* in the murine lung, it is not known whether the expression of individual allergen proteases is dependent on the type of substrate it encounters. Inhaled *Af* would initially contact the mucus layer of the host airway epithelium and as it grows, damage to this barrier would expose the underlying connective tissue and different substrates such as collagen and elastin. In the current study, the amount, type, and activity of major allergen proteases secreted by *Af* was determined in response to either physiological substrates such as lung homogenate and mucin or a defined protein substrate such as casein.

## Materials and methods

### Culture media

Vogel's salts (50×) were used as a base for all liquid media and prepared as previously described (Vogel 1956), with the omission of ammonium nitrate and chloroform. Vogel's salts (1×) were supplemented with bovine milk casein (1 % w/v; Sigma–Aldrich, Poole, UK), freeze dried ground porcine lung (0.4 % w/v; local abattoir) or porcine gastric mucin (1 % w/v; Sigma–Aldrich). In addition, Vogel's salts were supplemented with ammonium nitrate (25 mM) and d-glucose (55.5 mM) to prepare Vogel's minimal medium. All media preparations were autoclaved for 15 min. Liquid cultures were performed in 2 L conical flasks containing 500 ml of sterilized medium capped with a sponge stopper.

### *Aspergillus fumigatus* strain and culture

All cultures and culture media were prepared and handled under aseptic conditions. *Af* strain Af293 was used in all experiments and was a kind gift from the Mycology Reference Centre Manchester (Wythenshawe Hospital, Manchester, UK). *Af* spores were prepared by growth on Sabouraud Dextrose Agar (Oxoid, Basingstoke, UK), in a vented 75 cm^2^ tissue culture flask, for 72 h at 37 °C. Spores were harvested by gentle agitation in PBS 0.05 % Tween 20, filtered through four layers of sterile Whatman lens cloth to remove hyphal fragments and counted using a Neubauer haemocytometer. Liquid culture media were inoculated with 1 × 10^6^ conidia/ml and incubated for up to 72 h at 37 °C on a shaker at 320 rpm.

### Growth and pH measurement

Growth, pH, and protease activity were measured in *Af* cultures at 12, 24, 36, 48, and 72 h postinoculation. Rate of growth was measured as change in dry biomass by collecting 5 ml of liquid culture at set times and applying to a predried filter, washing with dH_2_O at 90 °C then drying at 110 °C for 72 h and reweighing. To analyse pH, 10 ml of liquid culture were collected and fungal biomass removed by filtration through a sterile J cloth and remaining supernatant sterilised by filtration using a 0.22 μm Corning CA Vacuum filter system and pH measured.

### Protease assays

The change in the level of protease activity in culture supernatant over 72 h was assessed in sterilized filtered supernatant using a resorufin-labelled casein assay according to the manufacturer's instructions (Roche Life Sciences, Welwyn, UK). The change in absorbance at 574 nm compared to a PBS blank was assessed using a microplate specrophotometer. For the calculation of rate of resorufin-labelled casein hydrolysis, supernatant from 48 h cultures was diluted to give a linear increase in absorbance over 60 min at 37 °C. Rate of change of absorbance was measured using linear regression and the protease activity calculated in International Units of protease activity per millilitre of enzyme solution (IU ml^−1^). One IU is defined as the amount of time in minutes taken to degrade 1 μmol of substrate. Protease activity in IU ml^−1^ was calculated using the Beer–Lambert equation using an extinction coefficient of 66 000 L mol^−1^ cm^−1^ for resorufin-labelled casein and normalized against dry biomass.

To characterize the class of protease activity present in culture supernatant, protease inhibitors were added to give final concentrations as follows: 2 mM PMSF (serine protease inhibitor; Sigma), 5 mM EDTA (metalloprotease inhibitor; Sigma), 10 μM E64 (cysteine protease inhibitor; Sigma), 25 μM Ilomastat (matrix metalloprotease inhibitor; Merck, Nottingham, UK) or 50 μM Pepstatin A (aspartic protease inhibitor, Merck, Nottingham, UK). Culture supernatants were preincubated with protease inhibitor for 30 min at room temperature prior to performing the assay. Culture supernatants treated with vehicle alone acted as negative controls.

### Size exclusion chromatography and SDS-PAGE

Culture supernatants were dialysed overnight at 4 °C against filter sterilised dH_2_O to remove low weight compounds using 10 kDa M.W.C.O snakeskin pleated dialysis tubing (Thermo Fisher Scientific, Basingstoke, UK). Dialysed supernatants were freeze-dried and stored at −20 °C until use. Supernatants were separated by size exclusion chromatography using a 24 ml Superdex S-200 column (GE Life Sciences, Little Chalfont, UK) in filter sterilised PBS (pH 7.4) at 0.5 ml min^−1^ and column flow through divided into 1 ml fractions using an AKTAPrime protein purification system (GE Life Sciences). Results were stored and viewed using PrimeView software and analysed using PrimeView Evaluate (GE Life Sciences).

Selected column fractions were concentrated using Corning 5 kDa M.W.C.O centrifugal concentration columns (Thermo Fisher Scientific) and samples (5 μl) run on 10 % Novex NuPage Bis-Tris Mini-gels in a MOPs Buffer system according to the manufacturer's instructions (Invitrogen, Life Technologies, Paisley, UK). Gels were stained using either Coomassie Brilliant Blue or silver nitrate staining according to the manufacturer's instructions (Bio-Rad, Hemel Hempstead, UK).

### Protein identification

For protein identification by mass spectrometry (MS), bands of interest were excised from the gel and dehydrated using acetonitrile followed by vacuum centrifugation. Dried gel pieces were reduced with 10 mM dithiothreitol, alkylated with 55 mM iodoacetamide, and then washed twice, alternately with 25 mM ammonium bicarbonate followed by acetonitrile, before drying by vacuum centrifugation. Samples were digested with trypsin overnight at 37 °C and then peptides extracted in one wash of 20 mM ammonium bicarbonate, and two of 50 % acetonitrile: 5 % formic acid. Tryptic peptides were dried by vacuum centrifuge to 20 μl and then analysed by LC-MS/MS using an Ultimate 3000 (LC-Packings, Dionex, Amsterdam, The Netherlands) coupled to an HCT Ultra ion trap mass spectrometer (Bruker Daltonics, Bremen, Germany). In brief, peptides were concentrated on a precolumn (5 mm × 300 μm i.d, LC-Packings) and then separated using a gradient from 98 % A (0.1 % FA in water) and 1 % B (0.1 % FA in acetonitrile) to 50 % B, in 40 min at 200 nL min^−1^, using a C18 PepMap column (150 mm × 75 μm i.d, LC-Packings). Data produced was searched using Mascot (Matrix Science, London, UK), against the SwissProt database and the UniProt database with taxonomy of fungi selected. Data was validated using Scaffold (Proteome Software, Portland, OR).

### RNA extraction and semiquantitative PCR

RNA from 48 h cultures was extracted using a FastRNA ProRed SPIN Kit (MP Biomedicals, Cambridge, UK). In brief, approximately 100 mg of fungal material was lysed using RNA Pro solution in Lysing Matrix C for 2 × 40 s in a FastPrep-24 instrument for 2 × 40 s at 4 m s^−1^ with the rest of the protocol performed according to the manufacturer's instructions. Eluted RNA was purified further using on column DNase digestion as part of the RNeasy RNA extraction kit (Qiagen, Crawley, UK). Quantitative real-time PCR using the Brilliant II SYBR Green QRT-PCR Master Mix, 1-Step Kit (Agilent Technologies, Wokingham, UK) was used to quantify the expression of allergen proteases, Mep, Alp1, and Lap1, relative to the housekeeping gene, β-tubulin, as described previously (Fraczek *et al.* 2010). Briefly, 25 μl reactions composed of 1× Brilliant II SYBR Green QRT-PCR Mix, 1 μM of each forward and reverse primer, RT/RNase block enzyme mixture, and 100 ng of total RNA were cycled in a DNA Engine Opticon thermocycler (Bio Rad) using the following reaction conditions, 1 h at 50 °C, followed by 40 cycles of 94 °C for 15 s, 52 °C for 30 s, and 72 °C for 75 s. Fluorescence was read at 52 °C three times in each cycle. In order to reduce background from potential DNA contamination primers were designed so that at least one of the primer sequences spanned an exon/exon boundary (indicated by *). The following primer pairs were used: Mep/Asp f 5 (AFUA_8G07080), sense (TACTCACGGTC*TTTCCAACCGAC), antisense (GCTTCAGACGGATGGCCGTC), Alp/Asp f 13 (AFUA_4G11800), sense (GAGCGCAGAC*GTTGCCCATG), antisense (CCTTGTGGGAAATGCTGCCCAG), Lap 1 (AFUA_3G00650), sense (AGCCCCGAGTTCATCCGA*AAGTC), antisense (GGCGTTTACGTGGGGCTGT).

### Statistical analysis

All statistical analysis was carried out using GraphPad Prism for Mac version 5.0b (GraphPad Software Inc, San Diego, USA).

## Results

### Growth of *Aspergillus fumigatus* on different protein substrates

**Fig 1:**
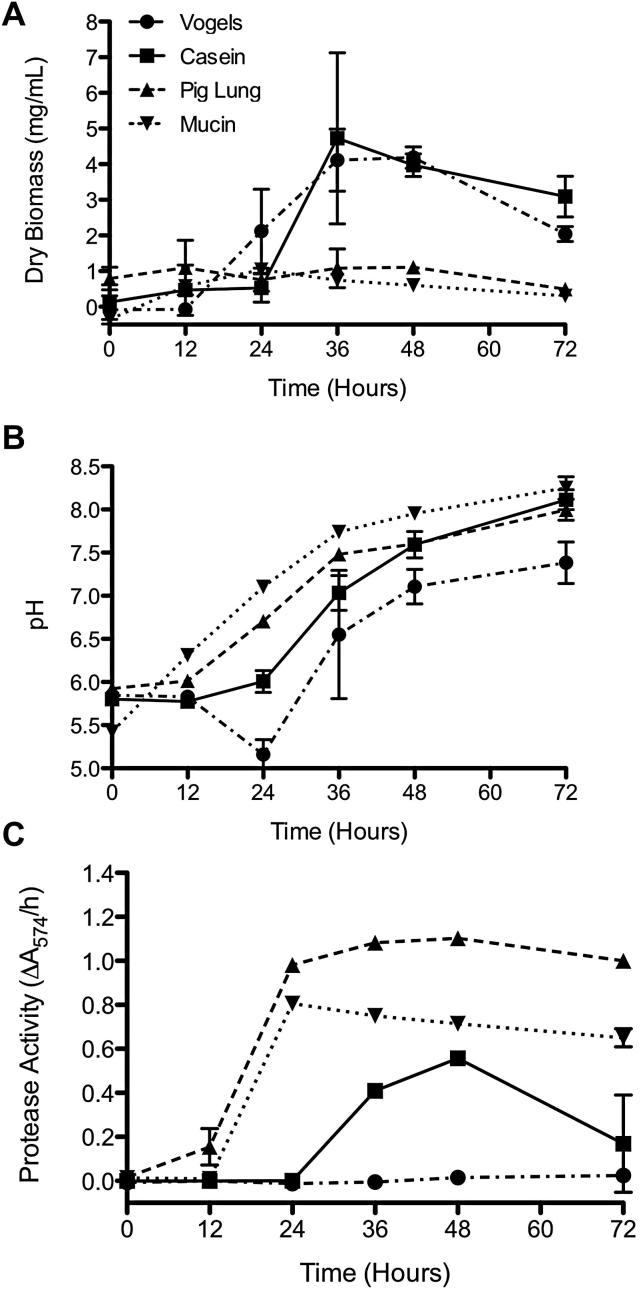
Protease activity during growth of *A. fumigatus* on different protein substrates. (A) Accumulation of dry biomass as an indication of *Af* growth, (B) change in pH, and (C) the level of protease activity was assessed in the four different *A. fumigatus* cultures over 72 h. Data represents mean ± SD and was analysed by two-way ANOVA with Bonferroni posttests (*n* = 3 biological replicates).

Initial studies investigated the rate of growth of *Af* in Vogel's medium, casein medium, pig lung medium, or mucin medium to determine whether different substrates regulate growth. The growth rate of *Af* was assayed by measuring dry biomass at 12, 24, 36, 48, and 72 h. The lag phase was similar in all cultures with little observable *Af* growth between 0 and 12 h (Fig 1A). *Af* exhibited similar growth characteristics in Vogel's medium and casein medium, with the only significant difference in growth seen at 24 h (*P* < 0.05). *Af* was found to grow more slowly in pig lung and mucin media compared to both casein and Vogel's media, with significantly lower levels of dry biomass at 36, 48, and 72 h (*P* < 0.05; Fig 1A). Maximum growth in Vogel's, casein, and pig lung media occurred between 36 and 48 h, however, in mucin medium, maximum growth occurred at 24 h.

In previous studies the pH of *Af* growth medium has been shown to change during growth and has been shown to be important in determining which proteases are secreted as well their stability and activity. In all cultures containing protein as the sole carbon and nitrogen source, pH of the growth medium gradually increased from around pH 6.0 to pH 8.0 during *Af* growth over 72 h (Fig 1B). In Vogel's minimal medium, the pH dropped between 0 and 24 h before beginning to increase. Casein and pig lung cultures exhibited a significantly higher pH than Vogel's minimal medium cultures between 36 and 72 h (*P* < 0.05), whilst mucin culture showed significantly higher pH between 12 and 72 h (*P* < 0.05). There were no significant differences between pH in mucin and pig lung cultures over time.

### Effect of different protein substrates on protease secretion by *Aspergillus fumigatus*

The induction of protease secretion over time by *Af* due to different culture media was assayed using a universal protease substrate at 0, 12, 24, 36, 48, and 72 h postinoculation. In supernatant from *Af* cultured in Vogel's minimal medium an increase in protease activity was observable but this was small compared with increases in protease activity in casein, pig lung, and mucin cultures. In supernatants from *Af* cultured in casein and pig lung media, protease activity increased over time and peaked at 48 h, whilst protease activity peaked at 24 h in mucin medium (Fig 1C). Protease activity in pig lung culture supernatants appeared earlier than in casein supernatants and was significantly higher at all time points. The changes in protease levels in casein, pig lung, and mucin culture supernatants tracked the growth of *Af*, with maximum protease levels detected at maximum growth in both pig lung and mucin cultures. Protease activity was not observed in uninoculated control culture media at any time point (data not shown).

**Fig 2:**
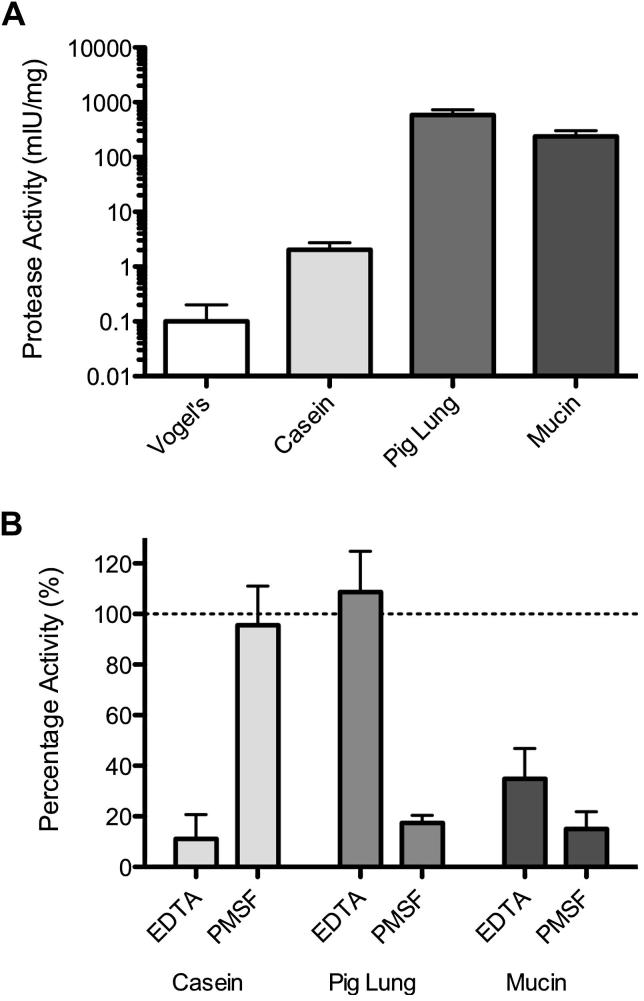
Characterisation of protease activity present in 48 h *A. fumigatus* cultures containing different protein substrates. (A) Rate of protease activity was determined in culture supernatant from 48 h cultures of *A. fumigatus*. Protease activity was normalised against dry biomass to account for differences in growth rate. (B) Inhibition of protease activity in culture supernatant from 48 h cultures of *A. fumigatus*. Percentage change in activity with inhibitor was relative to uninhibited control supernatant set at 100 % for each culture. Data represents means ± SD and was analysed by *t*-test, compared to Vogel's medium (*n* = 3 biological repeats).

In order to accurately quantify the levels of secreted protease activity, 48-h *Af* culture supernatants were diluted until a linear change in resorufin-labelled casein hydrolysis was observed by measuring absorbance at 574 nm over 60 min. Results were normalised against dry biomass to account for variations in protease amount due to differences in growth rate. Protease activity in pig lung culture supernatants was found to be greatest compared with mucin cultures (≈2 fold difference), casein cultures (≈300 fold difference), and Vogel's cultures (≈1000 fold difference, Fig 2A). This suggested that some media were more potent inducers of protease secretion than others; however the secretion of different proteases may lead to variation in the activity of secreted proteases.

To characterise the class of proteases secreted by *Af* during growth on different protease substrates, protease inhibitors were used to inhibit specific classes of protease and the protease activity measured. In all culture supernatants, E64 (cysteine protease inhibitor) and Pepstatin A (aspartic protease inhibitor) were not found to cause significant changes in protease activity (data not shown). Protease activity in casein culture supernatant was found to be inhibited predominantly by EDTA (11 % activity of uninhibited control), suggesting that metalloproteases were responsible for the majority of the observed protease activity (Fig 2B). Conversely protease activity in pig lung culture supernatant was mainly inhibited by PMSF alone (17 % activity of uninhibited control), suggesting that serine proteases are dominant within this culture supernatant. In mucin culture supernatant, proteases were partially inhibited by both EDTA and PMSF (35 % and 15 % activity of uninhibited control respectively), suggesting that both matrix metalloproteases and serine proteases were secreted.

Taken together these results suggest that the use of different protein substrates as the sole carbon and nitrogen source in culture medium affects both the growth of *Af* and the levels of protease activity observed in culture supernatants. It also appears to affect the class of protease activity present within the culture supernatant suggesting that different proteases are secreted or activated in culture supernatants in response to growth on different media.

### Identification of proteases secreted by *Af* during growth on different protein substrates

**Fig 3:**
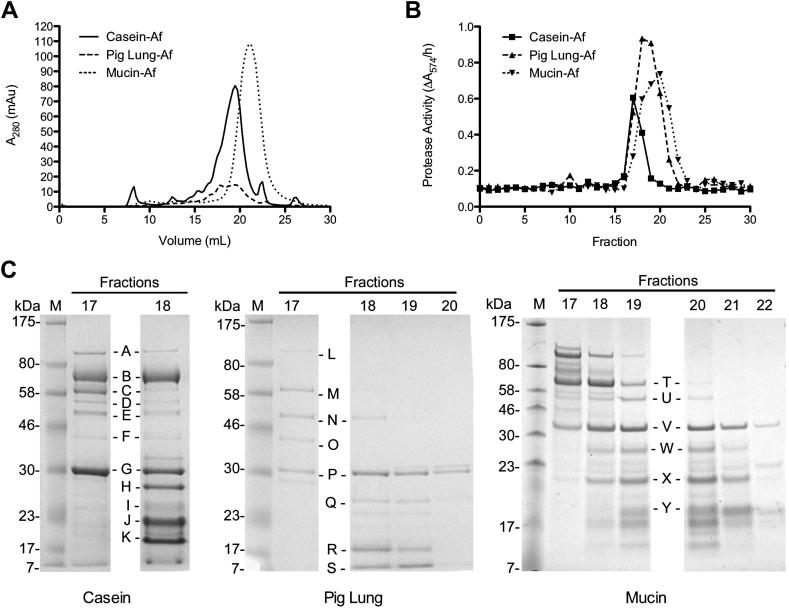
Identification of proteases secreted by *A. fumigatus* in response to different protein substrates. (A) Equal volumes of filtered and dialysed culture supernatant was subjected to size exclusion chromatography and protein levels measured continuously by UV absorbance at 280 nm. (B) Fractions collected at 1 ml intervals were analysed for protease activity. (C) SDS-PAGE of proteolytically active fractions showing bands analysed for protein identification by LC-MS/MS (see Table 1).

In order to analyse differences in protease secretion profiles of *Af* on different substrates, 48-h culture supernatants were analysed by size exclusion chromatography (Fig 3A). To identify the fractions containing protease activity, each 1 ml sample was assayed using resorufin-linked casein assay. Protease activity was detected in fractions 17 and 18 in casein culture supernatant, fractions 17–20 in pig lung culture supernatant, and fractions 16–20 in mucin culture supernatant (Fig 3B). To analyse the proteins present in proteolytically active fractions, samples were concentrated, proteins separated by SDS-PAGE and individual bands selected for identification by LC-MS/MS (Fig 3C and Table 1).

Comparing casein, pig lung, and mucin active fractions, several proteins were found to be common to both, including, β-d-glucoside glucohydrolase, Mannosidase MsdS, FAD-dependent oxygenase, and Cell wall β-1,3-endoglucanase (Table 1). These enzymes may be associated with cell wall modification and remodelling during growth and are expected during fungal growth *in vitro*.

*Af* was found to secrete different proteases dependent on the protein substrate present in the culture medium. Growth of *Af* in casein medium resulted in the secretion of the metalloproteases, aminopeptidase Y (Lap 1, Q5VJG5), and elastinolytic metalloprotease (Mep/Asp f 5, P46075) as suggested by inhibitor studies however the alkaline serine protease (Alp1/Asp f 13, P28296) was also present. Conversely, growth in pig lung medium resulted in the secretion of Alp1 as suggested by inhibitor studies but also Mep was detected, whilst growth in mucin medium resulted in the secretion of Alp1 but neither of the metalloproteases which was unexpected from inhibitor studies. These results again clearly demonstrate separate protease secretion profiles determined by different culture substrates.

### Gene expression of proteases by *Af* grown on different protein substrates

**Fig 4:**
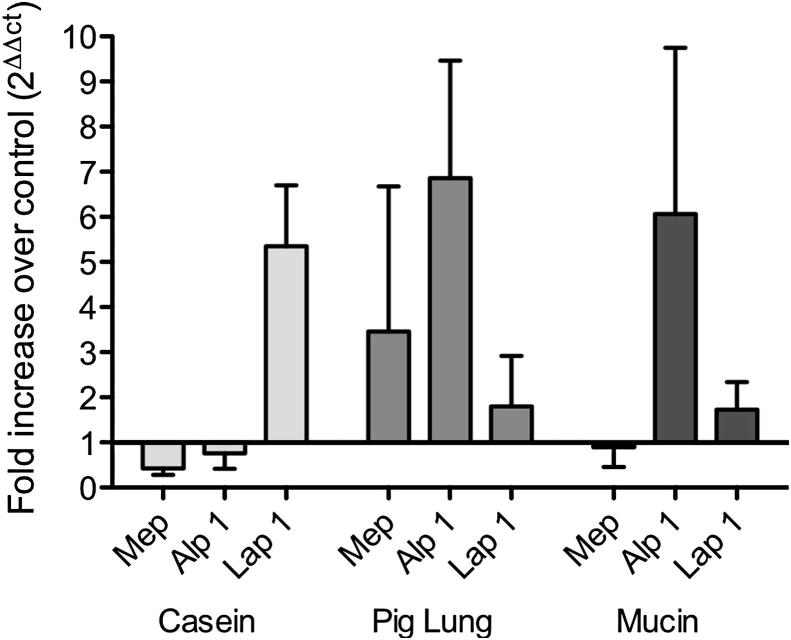
Protease gene expression in *A. fumigatus* cultures in response to different protein substrates. Protease gene expression was assayed by qPCR and expression of Mep, Alp 1, and Lap 1 was normalised against β-tubulin. Differences in gene expression in protein containing media compared with control Vogel's minimal medium were calculated using 2ΔΔct. Data represents means ± SD (*n* = 3 biological repeats) and was anaylsed by both one- and two-way ANOVA.

Gene expression of the proteases identified by LC-MS/MS was determined using qPCR at 48 h postinoculation in casein, pig lung, and mucin media and compared with Vogel's medium where no protease activity was detected. In casein cultures, Lap 1 expression was found to be significantly upregulated compared with Mep and Alp1 (Fig 4; *P* < 0.01). In pig lung cultures, both Mep and Alp1 gene expressions were found to be upregulated compared with Lap 1 whereas in mucin medium, only Alp 1 gene expression was found to be upregulated compared with Mep and Lap 1.

In pig lung and mucin cultures, gene expression of proteases (Alp 1 and Mep) correlated well with the protein detected in culture supernatant by mass spectrometry. However in casein cultures, Lap 1 appeared to be the only protease to show upregulated expression at 48 h whereas all three proteases (Alp 1, Mep, and Lap 1) could be detected in casein culture supernatant by mass spectrometry suggesting different regulatory mechanisms in the secretion of the proteases and regulation of gene expression. Furthermore, metalloprotease activity was demonstrated in mucin culture supernatant using inhibitors however only the serine protease, Alp 1 was shown to be upregulated and its protein detected by mass spectrometry suggesting that another MMP distinct from Lap 1 and Mep may be secreted and identified as an uncharacterized protein by mass spectrometry produced at low levels.

## Discussion

As saprotrophs, the ability of fungi to regulate and produce suitable enzymes to digest complex protein substrates in the environment is essential for successful colonisation of their surroundings (Denning *et al.* 2006). Furthermore, the mechanisms controlling the secretion of proteases by *Aspergillus fumigatus* are of particular interest, as proteases from *Af* and other *Aspergillus* species have been suggested to be involved in the development of allergic diseases such as SAFS and ABPA (Denning *et al.* 2006). Previous studies have suggested that the secretion of proteases by *Af* is regulated in response to pH and the availability of primary nitrogen sources (Bergmann *et al.* 2009; Sriranganadane *et al.* 2010) and that growth on a variety of protein only substrates including, serum and BSA (Gifford *et al.* 2002), collagen (Monod *et al.* 1993), elastin (Frosco *et al.* 1992), and fibrinogen (Larcher *et al.* 1992), results in the secretion of both serine and metalloproteases. In this current study, we aimed to investigate the effect of different protein substrates on the secretion of proteases by *Af*.

Results demonstrated that different protein substrates resulted in significantly different levels of protease secretion by *Af*. In particular, mucin and pig lung homogenate have a direct relevance to the type of physiological substrates that the fungus would encounter following inhalation. Furthermore, results also showed the differential secretion of three proteases, Alp 1, Mep, and Lap 1 by *Af* in response to changes in protein substrate explaining the dominance of different classes of protease activity in *Af* culture supernatants. Growth on casein medium, used as an alternative standard protein substrate, resulted in culture supernatants dominated by MMP activity and analysis of culture supernatants by LC-MS/MS revealed the presence of metalloproteases, Lap 1, and Mep. The serine protease, Alp 1, was also shown to be present in the culture supernatant by LC-MS/MS; however, there appeared to be little detectable serine protease activity present following addition of serine protease inhibitors. This suggests that Alp 1 may not have been secreted in sufficient quantities to be detected in protease assays, or that the secreted Alp 1 was inactive. Analysis of Lap 1, Alp 1, and Mep transcription by qPCR revealed that Lap 1 was strongly upregulated compared with Alp 1 and Mep, which were both downregulated. Taken together these results suggest that casein medium caused the transcriptional upregulation of Lap 1 resulting in a culture supernatant dominated by metalloprotease activity. The presence of Mep and Alp 1 in the supernatant, despite downregulation of expression, may be due to temporal regulation of their transcription, and it is possible that they may have been expressed prior to sampling at 48 h.

In contrast, growth in pig lung medium resulted in culture supernatant dominated by serine protease activity as shown by inhibitor studies. Analysis of protease containing fractions of pig lung culture supernatant by LC-MS/MS, demonstrated the presence of both Alp 1 and Mep, whilst analysis of gene transcription showed that transcription of Alp 1 was strongly upregulated at 48 h. Taken together, these results suggest that the serine protease, Alp 1, is strongly upregulated during growth in pig lung medium and is responsible for the serine protease activity observed in these cultures. The presence of Mep in culture supernatants by LC-MS/MS without detectable metalloprotease activity may again be because Mep was not secreted in sufficient quantities to be detected in the protease assay, or that the secreted Mep was in an inactivate form.

Analysis of mucin cultures, with addition of protease inhibitors, revealed that protease activity was due to both metalloproteases and serine proteases. However, detection of proteases by LC-MS/MS only revealed the presence of the serine protease, Alp 1. Furthermore, analysis of transcription revealed that only Alp 1 was significantly upregulated. Together these results suggest that Alp 1 was the main protease responsible for the serine protease activity detected in mucin culture supernatant. The metalloprotease activity that was found to be present by inhibitor studies was not detected as Mep or Lap 1 by LC-MS/MS and transcription of these proteases was not upregulated at 48 h. This may indicate the presence of novel proteases with MMP activity that still needs to be isolated and identified in mucin culture supernatant. The degradation of mucins by *Af* has been reported previously (St Leger & Screen 2000), however, the proteases involved were only identified by their catalytic activity and suggested to belong to the aminopeptidase, subtilisin, and trypsin-like protease families. Taking the study as a whole, the results obtained for the different experiments are partly inconsistent. For instance, results suggest that in casein and pig lung culture supernatants, Alp1 and Mep, respectively, were found to be present by mass spectrometry, but were not found to be active during inhibitor studies. The presence of protease without activity suggests that proteases were either, secreted in different amounts in the supernatant, not activated following secretion, or inactivated or inhibited in the supernatant. Given the presence of propeptide domains present in both Mep and Alp1, it is possible that proteases may have been secreted into the culture supernatant but not activated. However in both cases the proteins present in the supernatants had a much lower molecular weight than those of the full translation of the open reading frame (ORF) of the protein, which implies posttranslational modifications involving cleavage of the propeptide/chaperone domain had already occurred. Alternatively, proteins may have been inactivated posttranslationally due to cleavage by other proteases in the cell cytoplasm, during secretion or in the culture supernatant. In casein and pig lung culture supernatants, the fragmentation patterns of Alp1 were slightly different. Both supernatants contained a 30 kDa Alp1 protein fragment however in casein culture supernatants only a 28 kDa fragment was present, whilst pig lung culture supernatants contained 25 kDa and 7 kDa fragments. It may be possible that differential cleavage or degradation of the Alp130 kDa fragment resulted in active and inactive forms of the protease. Finally *Af* has been shown to secrete an elastase inhibitor, AFUEI (AFUB 034300), which strongly inhibits *Af* elastinolytic activity (Okumura *et al.* 2008). This inhibitor may be secreted by *Af* under certain growth conditions and bind selectively to Alp1 resulting in inhibition.

Regulation of the production of protease *Af* may have been due to differences in transcription of the protease genes. In the current study, levels of allergen protease mRNA were measured by qPCR, giving an indication of the levels of gene transcription. However, posttranscriptional and posttranslational events may have resulted in different levels of protein secretion. Measurement of the baseline levels of Mep and Alp1 mRNA indicated that expression of these genes was significantly lower than the housekeeping gene β-tubulin. This matched the results of Fraczek *et al.* (2010) where levels of Mep and Alp1 mRNA were also found to be considerably lower than β-tubulin. Taken together with the data for protease activity in culture supernatants, results from this study suggest that the levels of Mep, Alp1, and Lap1 found in culture supernatants may be regulated at the transcriptional level. Possible mechanisms for the secretion and regulation of protease activity within *A. fumigatus* culture supernatants have been proposed. For instance, evidence suggests that several proteases, including Mep and Alp1 are regulated by the transcription factor prtT (Bergmann *et al.* 2009; Sharon *et al.* 2009), potentially through the control of the CpcC eIF2α sensor kinase, which detects environmental stresses such as amino acid depletion (Krappmann & Braus 2005). Results from this study suggest that Mep and Alp1 were coregulated by the same transcription factor, but a novel result suggests that Lap1 was regulated by a separate transcription factor, as levels of Lap1 expression in casein cultures appeared to be increased relative to Mep and Alp1. It is also possible that transcription may have not resulted in translation, and therefore measurement of Mep, Alp1, and Lap1 levels in the culture supernatants would confirm the results shown by qPCR.

Bioinformatic studies suggest that the *Af* genome encodes over 100 proteases and 26 nonpeptidase homologues of which between 47 are predicted to be secreted extracellularly (Watson *et al.* 2011). Although the current results are similar to those shown in other studies where Mep, Lap 1, and Alp 1, were shown to be secreted by *Af* during growth on lung structural components including, collagen and elastin (Frosco *et al.* 1992; Kolattukudy *et al.* 1993; Monod *et al.* 1993; Wartenberg *et al.* 2011), additional proteases have also been detected including aspartic protease (pep1), sedolisins (SedA-D), aegerolysins (Asp-haemolysin), and dipeptidyl peptidases (Dpp IV and V). There are a number of reasons why some of these other proteases were not detected in the current study. Firstly, the pH of the culture media may not be inductive to the secretion of some proteases. For instances, Sedolisins are active under acidic conditions (Reichard *et al.* 2006) whereas the pH of the Af cultures in the present study became more alkaline over time. Another reason may be due to the method used to identify proteases in the current study, in the sense that it only identified protease activity capable of cleaving the resorufin-labelled casein substrate used in the protease assay and only fractions that showed degradation of this substrate were analysed further by LC-MS/MS. Therefore, some types of secreted proteases that have been identified in *Af* culture supernatant under neutral pH conditions by others, such as the exopeptidase Dpp V (Neustadt *et al.* 2009; Singh *et al.* 2010; Wartenberg *et al.* 2011) may not be detected as they may have been in fractions that were not analysed which is a limitation of this study. A previous study by Sriranganadane *et al.* (2010) used a shotgun mass spectrometry method which identified all proteins present in the *Af* culture supernatants without the need to first identify proteolytically active fractions and allowed for some quantification of the amount of protease present. Neustadt *et al.* (2009) characterized and identified secreted proteases in *Af* culture supernatant using free flow electrophoresis with protease activity in each fraction assayed using specific fluorescently labelled reporter peptides. Fractions with high protease activity were further subjected to LC-MS/MS analysis for protease identification and showed that mainly metalloproteases and serine proteases are involved in the degradation of reporter peptides although no quantitative profile was described. The use of these alternative proteomic profiling techniques in future studies might be used to identify all of the proteases present in supernatants from complex protein substrate cultures. Furthermore, the development of specific antibodies against these proteases will enable protein levels in culture supernatants to be more accurately quantified by western blot or ELISA.

It has been suggested that proteases from *Af*, specifically the allergen proteases Mep and Alp 1, might be involved in the development of allergy either directly *via* the protease dependent release of proinflammatory cytokines (Borger *et al.* 1999; Kauffman *et al.* 2000) or indirectly as adjuvants causing sensitisation to other allergens (Kurup *et al.* 2002). In this context, a pathway that suppresses the expression of certain proteases may be a potential target for future drug therapies thereby reducing lung inflammation during exposure and colonisation of the lungs in individuals with SAFS and ABPA. Furthermore, if *Af* proteases are important in the development of allergy and inflammation, the results of this study suggest that *Af* extracts used in both clinical diagnostics and research studies should be accurately defined and fully characterised. A report by Esch (2004) showed that there was large amounts of variation in the protein and carbohydrate composition of commercially available *Alternaria alternata* extract. A further study by Aas *et al.* (1980) found that different preparations of fungal allergens, from the same species of fungi, including *Aspergillus*, provided by different manufacturers were found to elicit different skin test results in the same patient. Findings from these studies and the results from this current study suggest a clear need for the standardization of the preparation of protease containing allergen extracts from *Af* and other fungi.

## Figures and Tables

**Table 1:** Proteins identified by LC-MS/MS in *A. fumigatus* culture supernatants. The SDS-PAGE bands are shown in Fig 3C and the number of peptide matches for each protein is shown. A confident match was considered to be any protein with three or more matched peptides. Proteases are highlighted in bold. Protein molecular weights are quoted from UniProt for protein translated from the full ORF.

Identified proteins	Molecular weight (kDa)	CADRE accession number	UniProt accession number	Protein matches (band and number of matched peptides)		
Casein medium	Pig lung medium	Mucin medium
Exo- β −1,3-glucanase	84	AFUA_6G13270	Q4WLJ9	–	L (3)	–
β-d-glucoside glucohydrolase	78	AFUA_7G06140	Q4WGT3	A (18)	L (12)	T (5) V (3)
**Aminopeptidase Y (Lap 1)**	**54**	**AFUA_3G00650**	**Q5VJG5**	**B (13)** **C (6)** **D (5)**	–	–
Mannosidase MsdS	54	AFUA_1G14560	Q6PWQ1	C (14)	M (14)	U (8)
FAD-dependent oxygenase	55	AFUA_3G00840	Q4WFW0	C (11)	M (9)	U (7)
Oxidoreductase, FAD-binding	50	AFUA_2G14480	Q4X072	C (4)	M (4)	–
Uncharacterised protein	49	AFUA_4G03200	Q4W9Z5	D (12)	N (10)	–
Glucooligosaccharide oxidase	51	AFUA_6G14340	Q4WL94	D (5)	–	–
**Elastinolytic metalloproteinase (Mep/Asp f 5)**	**69**	**AFUA_8G07080**	**P46075**	**E (5)**	**N (5)**	–
Cell wall β-1,3-endoglucanase	45	AFUA_3G00270	Q4WG16	F (7)	O (4)	V (4)
Class V chitinase	46	AFUA_3G07160	A4D9F7	F (3)	N (6)	–
Uncharacterised protein	37	AFUA_5G01120	Q4WDY6	F (4)	–	–
Uncharacterised protein	37	AFUA_8G00630	Q4WB08	–	P (5)	–
**Alkaline serine protease (Alp1/Asp f 13)**	**42**	**AFUA_4G11800**	**P28296**	**G (8)** **H (4)**	**P (6)** **Q (3)** **S (5)**	**V (5)** **W (5)** **X (3)** **Y (3)**
Chitosanase	25	AFUA_4G01290	Q875I9	H (9)	–	–
Uncharacterised protein	33	AFUA_5G10930	Q4WV60	H (4)	–	–
Cell wall protein PhiA	19	AFUA_3G03060	Q4WF87	I (3) J (3)	–	–
Cell wall protein	19	AFUA_3G01130	Q4WFT1	K (12)	R (8)	X (9)
Cu/Zn Superoxide Dismutase	16	AFUA_5G09240	Q9Y8D9	K (4)	–	–
Allergen Asp f 15	16	AFUA_2G12630	O60022	–	–	X (3)
Secreted antimicrobial peptide	10	AFUA_8G00710	Q4WB16	–	–	Y (3)
